# Pathological Mechanisms of Tendinopathy Pain: From Mechanical Overload to Neuroimmune–Vascular Crosstalk

**DOI:** 10.1155/prm/1712265

**Published:** 2026-09-16

**Authors:** Chenyu He, Aikang Li, Qingyi Liu, Mingyang Xu, Hongbo You, Hewei Li

**Affiliations:** ^1^ Department of Orthopedics, Liyuan Hospital, Tongji Medical College, Huazhong University of Science and Technology, Wuhan 430000, Hubei, China, hust.edu.cn; ^2^ Department of Orthopedics, Tongji Hospital, Tongji Medical College, Huazhong University of Science and Technology, Wuhan 430077, China, hust.edu.cn

**Keywords:** central sensitization, ion channels, mechanotransduction, neoinnervation, neuroimmune coupling, neuroplasticity, pain mechanisms, peripheral sensitization, tendinopathy

## Abstract

Characterized by localized pain and functional impairment, tendinopathy remains a significant challenge in sports medicine. Traditional models of structural degeneration and inflammation inadequately explain the clinical discordance between tissue damage and pain severity. This review explores the paradigm shift in understanding tendinopathic pain, elucidating the transition from a simple mechanical stress model to a complex neuroplastic disorder. We narratively review the multilayered pathophysiological mechanisms underlying chronic tendinopathic pain. Specifically, we outline abnormal mechanical load transduction, peripheral neuroimmune–vascular coupling, and central nervous system (CNS) remodeling. Abnormal mechanical overload initially activates mechanosensitive ion channels, transducing physical stress into biochemical nociceptive signals. Subsequently, chronic immune responses and neurovascular coupling drive peripheral sensitization, which is further amplified by aberrant sensory neoinnervation and neuroimmune crosstalk. Furthermore, CNS involvement manifests as spinal‐level central sensitization and maladaptive remodeling of the motor cortex. Managing chronic tendinopathic pain requires transcending the traditional focus on localized structural lesions. Future strategies should prioritize precise clinical phenotyping, targeted pharmacotherapy, and neuroplasticity‐informed rehabilitation to optimize patient outcomes.

## 1. Introduction

Tendinopathy remains one of the most challenging conditions in sports medicine and musculoskeletal rehabilitation [1–4]. Its primary clinical features include localized pain, point tenderness, and load‐dependent functional impairment. Tendon injuries account for a significant proportion of sports and musculoskeletal disorders, severely impairing physical function and quality of life. With an estimated 30 million cases annually worldwide, these injuries impose a substantial socioeconomic burden through lost productivity and healthcare costs [5]. Although interventions such as physical therapy, nonsteroidal anti‐inflammatory drugs (NSAIDs), and injections are widely used, therapeutic outcomes frequently remain unsatisfactory. This clinical dilemma stems primarily from an incomplete understanding of the precise neurobiological mechanisms underlying tendinopathic pain. Historically, clinicians and researchers have relied on classic inflammatory models to explain this pain [6, 7]. The early term “tendinitis” implied an acute inflammatory origin, while the subsequent shift to tendinosis emphasized abnormal collagen fiber alignment and extracellular matrix (ECM) degeneration [8–10]. However, extensive clinical and radiological evidence now challenges this structural paradigm. Studies demonstrate a highly inconsistent relationship between tendon pain and structural damage. Severely degenerated tendons often appear asymptomatic on imaging, whereas structurally normal tendons can exhibit severe pain [10]. This discordance indicates that structural impairment does not directly equate to nociception, highlighting the urgent need to redefine the etiology of tendinopathic pain.

Consequently, the pathophysiological model of tendinopathic pain is shifting from pure mechanical strain toward inflammatory mechanisms and neurovascular coupling. Ackermann et al. noted that early tendinopathy frequently involves aberrant cellular‐level inflammation and failed healing responses [11]. Building on this, Gehwolf et al. proposed a “tendon pathological cycle,” highlighting the dynamic, mutually amplifying interplay among mechanical stress, inflammation, and angiogenesis [12]. Aberrant mechanical loading triggers cellular catabolism and the release of inflammatory mediators via Piezo1 channels and integrins. These mediators lower the mechanosensory threshold of tenocytes and upregulate vascular endothelial growth factor (VEGF), promoting pathological neovascularization [13–15]. Crucially, aberrant alterations within the peripheral nervous system have emerged as a primary research focus. In chronically painful tendons, nociceptive nerve fibers accompany neovessels, infiltrating the typically avascular and aneural tendon body and resulting in aberrant nerve sprouting [16, 17]. This nociceptor proliferation prompts a substantial release of local pronociceptive neuromediators, driving robust neurogenic inflammation and peripheral sensitization.

However, peripheral mechanisms alone cannot fully explain the widespread mechanical hyperalgesia and motor control deficits observed in tendinopathy. Consequently, recent research has pivoted toward the neuroplastic remodeling of the central nervous system (CNS). A systematic review and meta‐regression analysis of multiple clinical trials demonstrated that central pain sensitization is highly prevalent among these patients [16]. As emphasized by Jayaseelan et al., evaluating CNS somatosensory alterations using standardized tools like quantitative sensory testing (QST) has emerged as a cutting‐edge frontier [18]. Under pathological conditions, continuous peripheral nociceptive input activates spinal glial cells, pathologically enhancing synaptic transmission within the dorsal horn. Furthermore, this sustained input induces maladaptive remodeling of the primary motor cortex, manifesting as augmented intracortical inhibition and disrupted higher‐order motor drive [19, 20].

In summary, chronic tendinopathic pain should no longer be viewed merely as a localized mechanical symptom. Instead, it must be redefined as a complex neuroplastic disorder initiated by aberrant mechanotransduction, amplified by neuroimmune crosstalk, and ultimately governed by profound CNS involvement. Based on this contemporary paradigm, this article systematically reviews the pathophysiological mechanisms driving the evolution of tendinopathic pain. We initially explore microscopic mechanical load transduction and peripheral neuroimmune coupling, subsequently progressing to spinal‐level central sensitization and microenvironmental dysregulation. Finally, we examine neuroplastic adaptations in the motor cortex and their implications for modern rehabilitation strategies, aiming to provide a comprehensive theoretical framework for the precision assessment and targeted therapy of tendinopathy.

### 1.1. Literature Search Strategy

This narrative review was informed by targeted searches of PubMed and Web of Science from database inception to August 2026, using combinations of terms related to tendinopathy, tendon pain, mechanical loading, mechanotransduction, ion channels, inflammation, neurovascular coupling, peripheral sensitization, central sensitization, and rehabilitation. We prioritized human clinical, imaging, histological, and translational studies of tendinopathy. Animal, in vitro, and nontendon studies were included only when they provided mechanistic context and are interpreted as indirect evidence rather than direct proof in human tendinopathic pain.

## 2. Mechanisms

Abnormal mechanical loading can initiate tenocyte and sensory‐terminal mechanotransduction, inflammatory mediator release, and ECM remodeling. These local changes may promote neurovascular remodeling and neuroimmune crosstalk, leading to peripheral sensitization; persistent nociceptive input may then contribute to spinal central sensitization and cortical and motor remodeling, culminating in chronic pain and motor dysfunction.

### 2.1. Structural and Mechanoadaptive Foundations of Tendons

#### 2.1.1. Histological and Biomechanical Hallmarks of Healthy Tendons

**FIGURE 1:**
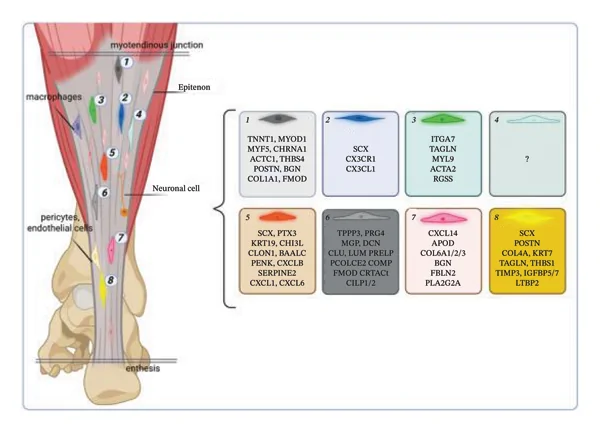
This figure delineates the specific cell subpopulations within the tendon microenvironment. Besides readily identifiable endothelial cells, macrophages, and neural cells, several other tendon‐resident populations exist. These are currently classified as distinct stromal and tenocyte subpopulations. Reproduced with permission from Ref. [12]. Copyright 2025 The Authors.

Tendons are highly organized connective tissues composed predominantly of tenocytes and the ECM. Constituting the bulk of the tendon, the ECM is rich in collagen (predominantly Type I, ∼95%), which accounts for 60–85% of its dry weight. These collagen molecules assemble hierarchically from nanoscale fibrils into microscopic bundles, ultimately forming the macroscopic tendon structure. This dense, parallel alignment along the longitudinal axis confers extraordinary tensile strength [21–23]. Tenocytes, functioning as mature fibroblast‐like cells, orchestrate the synthesis of all ECM constituents. Recent single‐nucleus and spatial transcriptomic analyses of healthy human hamstring tendons have delineated distinct cellular populations, confirming the presence of Mkx+ and Pdgfra + fibroblast subpopulations. Beyond these classic fibroblasts, this profiling revealed previously undocumented cell clusters within tendon tissue, including skeletal muscle cells, adipocytes, satellite cells, and an undefined neural lineage, alongside known endothelial, mural, and immune cells (Figure 1) [24]. Positioned end‐to‐end between collagen fibers, tenocytes communicate intercellularly via gap junctions, facilitating the rapid transit of ions and small molecules [25, 26]. This connectivity is dynamically modulated by pH, calcium gradients, and mechanical loading—factors paramount for tendon homeostasis and adaptive capacity [27, 28]. Furthermore, the ECM harbors noncollagenous components, such as small proteoglycans (PGs)and glycoproteins, which intercalate with the collagen network to synergistically regulate tendon biomechanics and the cellular microenvironment. The macroscopic tendon is ensheathed and internally permeated by auxiliary connective tissues. It also features highly specialized architectural adaptations at interfaces like the myotendinous junction (MTJ) and the osteotendinous junction (OTJ) [29].

Consequent to their hierarchical architecture, tendons exhibit defining biomechanical properties—namely, high mechanical strength and viscoelasticity—enabling them to robustly bear and transmit loads. Furthermore, tendons are inherently mechanoresponsive, adaptively remodeling their structural and functional integrity in response to fluctuating mechanical demands. While physiological loading promotes tendon homeostasis, mechanical overload or complete disuse precipitates deleterious changes. This remarkable mechanical adaptability is fundamentally driven by the mechanosensory capacity of resident tenocytes.

#### 2.1.2. Peripheral Tissue Alterations and the Substrate of Pain Genesis

Microscopic evaluation of painful tendon biopsies reveals distinct histopathological hallmarks. These include collagen disarray, fiber segregation, elevated PG and water content, neovascularization, sensory hyperinnervation, and cellular hyperplasia [30–33]. Under physiological conditions, tendon collagen fibers exhibit a highly ordered, parallel architecture. In contrast, tendinopathic tissues demonstrate overt architectural disruptions, manifesting as irregular crimping, laxity, and exaggerated waviness. Concurrently, there is a significant upregulation of Type III collagen (COL‐III), an isoform predominantly expressed during tissue repair [9, 10, 32].

These pathological features should be interpreted within a stage‐ and site‐aware framework. In the continuum model, reactive tendinopathy is mainly characterized by a relatively homogeneous cellular and matrix response, with increased PG‐bound water and limited collagen disruption. Tendon dysrepair shows greater collagen separation, matrix disorganization, and heterogeneous cellularity. Degenerative tendinopathy displays more marked collagen disarray, focal cell loss, and extensive matrix, vascular, and neural remodeling [33]. Yet these patterns vary by anatomical site. Achilles and patellar tendons commonly experience repetitive tensile loading, whereas lesions of the rotator cuff and lateral epicondyle may additionally be influenced by compression, insertional anatomy, or age‐related changes [17, 21, 30, 34]. Accordingly, histopathological evaluation should integrate structural markers, including COL‐I and COL‐III, metalloproteinases (MMPs), and ADAMTS, inflammatory‐cell markers, including CD68 and tryptase, vascular markers, including CD31 and VEGF, and neural and nociceptive markers, including PGP9.5, substance P, calcitonin gene–related peptide (CGRP), and NGF, while recognizing that their expression is not uniform across stages or sites [35–39].

Nociception in tendinopathy likely stems from functional neural adaptations rather than macroscopic shifts in global innervation patterns. Although sensory nerve density increases in pathological tendons, pain genesis is more intimately linked to terminal nerve sprouting, central sensitization, or the conditional activation of non‐nociceptive afferents, such as Aβ fibers [40]. Furthermore, a persistent, low‐grade inflammatory milieu characterizes chronic tendinopathy. This environment features the infiltration of macrophages and mast cells, alongside the upregulation of proinflammatory mediators like substance P and prostaglandin E2 (PGE2). These mediators propagate nociception by sensitizing nerve terminals and augmenting vascular permeability [34, 41].

Consequently, tendinopathic pain is not merely a direct manifestation of an isolated structural lesion. Instead, it results from synergistic interactions among structural deterioration, neural dysfunction, and an altered local biochemical microenvironment. This mechanistic complexity dictates that clinical rehabilitation must judiciously balance mechanical load management with nociceptive modulation, thereby preventing relapse induced by premature high‐intensity training [42].

To complement qualitative morphological observations, quantitative histopathological analysis can provide objective measures of tendon neurovascular remodeling. In digitally acquired immunohistochemical images, CD31‐positive microvessel density and PGP9.5‐positive nerve‐fiber density can be quantified per unit tissue area, whereas integrated optical density (IOD) may be used to semiquantitatively assess the immunoreactivity of nociceptive mediators such as CGRP and substance P. Meaningful comparisons require standardized sampling regions, staining procedures, image‐acquisition settings, and normalization to tissue area. These quantitative measures should be interpreted together with clinical pain phenotypes rather than as direct surrogates for pain severity [17, 30, 43].

### 2.2. Mechanical Strain, Metabolic Dysregulation, and Microenvironmental Factors

**FIGURE 2:**
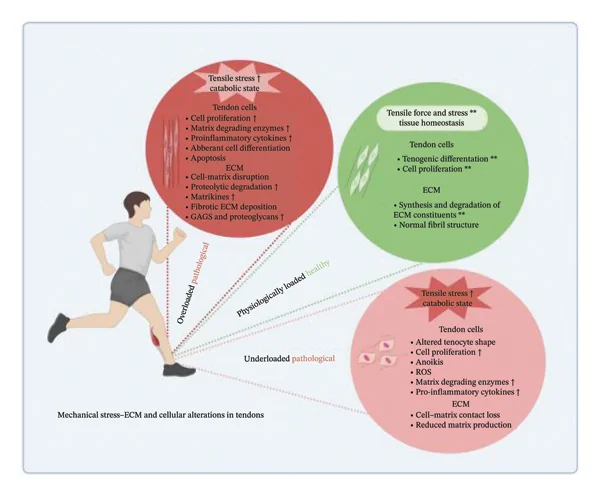
Schematic diagram illustrating the core pathophysiological alterations in tenocytes and the extracellular matrix induced by mechanical overloading and underloading. Reproduced with permission from Ref. [12]. Copyright 2025 The Authors.

Building upon these structural and neural complexities, recent research demonstrates that mechanical overload, manifesting as repetitive tensile strain [44, 45], abrupt load escalation [33], or compression [46], serves as the primary catalyst initiating the tendinopathic pain cascade. While physiological loading promotes anabolic adaptation, repetitive loading, mechanical overload, or complete disuse precipitates catabolic responses, ultimately driving pathogenesis (Figure 2) [47, 48]. Central to this pathogenesis is the mechanosensory detection of aberrant forces by resident tenocytes and sensory nerve terminals, which subsequently transduce these physical stimuli into sustained nociceptive signals. This mechanotransduction, the process by which cells detect and convert mechanical stimuli into biochemical responses, relies heavily on the cytoskeleton. Acting as a mechanotransductive conduit, the cytoskeleton structurally couples external mechanical inputs with intracellular mechanosensors [12]. Tenocytes rapidly respond to mechanical perturbations, such as tensile or shear stress, by dynamically remodeling their actin cytoskeleton. This reorganization modulates sensor proteins localized at the plasma membrane and within the nucleus, translating mechanical cues into intracellular signaling pathways that govern gene expression and post‐translational modifications [49, 50].

At the membrane level, ion channels respond to voltage, mechanical, or chemical fluctuations by modulating ionic fluxes. In tenocytes, these channels regulate critical functions, including calcium signaling, osmoregulation, maintenance of the resting membrane potential, and mechanical stimulus detection [51]. Anchored to both the cytoskeleton and ECM, these channels are structurally primed for direct gating via mechanical deformation. For instance, Piezo1, an archetypal mechanosensitive channel, is expressed in tendon and has been implicated in tenocyte mechanotransduction. Matrix tensile or shear stress physically deforms the plasma membrane, directly gating Piezo1 and triggering a transient calcium (Ca^2+^) influx. This influx sequentially activates downstream pathways to preserve biomechanical homeostasis [52]. Because mechanical loading frequently alters localized osmolarity, the transient receptor potential vanilloid 4 (TRPV4) channel detects these combined mechanical and osmotic fluctuations, orchestrating cell volume and matrix metabolism via calcium regulation [53].

Crucially, under pathological overload, these channels function as molecular sensors that directly translate aberrant mechanical stress into nociceptive signals. A diverse array of mechano‐ and chemosensitive channels, including stretch‐activated channels (SACs) [54], acid‐sensing ion channels (ASICs) [55], and voltage‐operated calcium channels (VOCCs) [51], is robustly expressed on both resident tenocytes and infiltrating sensory nerve terminals.

Building on this mechanotransductive framework, a self‐reinforcing biomechanical feedback loop may help explain why mechanosensitive signaling can persist during apparently physiological loading. Inflammatory mediators and MMP activity can degrade and disorganize the collagen–PG network, thereby altering tendon mechanical properties and local cell–matrix interactions [12, 36–38, 44]. Under the same external load, this heterogeneous matrix may concentrate strain at selected cell–matrix interfaces and sensory nerve endings [49, 50]. Repeated activation of mechanosensitive channels can then sustain calcium‐dependent catabolic and inflammatory signaling, further aggravating ECM remodeling [52, 56, 57].

#### 2.2.1. Mechanosensitive Ion Channels (MSCs)

**FIGURE 3:**
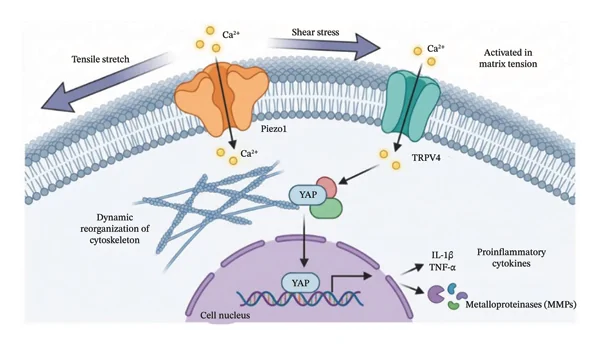
Schematic representation of mechanosensitive ion channel–mediated mechanotransduction and the subsequent inflammatory cascade in tenocytes. Tensile and shear stresses activate Piezo1 channels localized on the plasma membrane, whereas TRPV4 channels are concurrently gated by extracellular matrix tension. The activation of both channels facilitates the intracellular influx of calcium ions. The subsequent surge in intracellular Ca^2+^ levels instigates dynamic cytoskeletal remodeling and promotes the nuclear translocation of the YAP protein complex. Within the nucleus, YAP interacts with DNA to upregulate gene transcription, ultimately culminating in the secretion of matrix metalloproteinases (MMPs) and proinflammatory cytokines, including IL‐1β and TNF‐α.

The activation of MSCs depends on conformational transitions between closed and open states, a process directly driven by local membrane tension [58, 59]. Repetitive or abrupt mechanical strain on pathological tendons directly activates these channels, mediating cation influx. This influx induces membrane depolarization, subsequently triggering intracellular nociceptive and proinflammatory signaling cascades (Figure 3). For instance, the TRPV1 channel functions as a multimodal nociceptive hub, integrating mechanical stretch with thermal, acidic, and endogenous proinflammatory cues [60]. Additionally, diverse MSCs, including SACs, collectively orchestrate the detection and transduction of noxious stimuli [61, 62].

Recent investigations into specific pathological microenvironments further illuminate the metabolic specificity of mechanotransduction. Notably, in diabetic tendinopathy models, mechanical stretch under hyperglycemic conditions initiates cellular calcium influx, subsequently activating the CaMKK2/AMPK signaling axis. This cascade drives focal adhesion kinase (FAK)–mediated cytoskeletal remodeling and upregulates early growth response 1 (EGR1), a protein critical for preserving the tenocyte phenotype [56]. These findings highlight the pivotal role of mechanical signaling in preserving cellular homeostasis and counteracting metabolic stress.

Holding significant translational value, the gating kinetics of SACs provide a precise mechanistic rationale for the hallmark load‐dependent nature of tendinopathic pain. Clinically, tendinopathic pain exacerbates with mechanical loading and subsides upon unloading. Furthermore, the positive correlation between pain intensity and load magnitude may partially explain the biophysical paradigm where channel open probability increases at higher membrane tensions. Additionally, the warm‐up phenomenon observed during exercise, characterized by pain attenuation during sustained activity, may correlate with ion channels entering a state of mechanodesensitization or functional saturation under continuous stimulation [63].

Pharmacological interventions targeting this mechanotransductive machinery represent a potential therapeutic direction. Preclinical studies suggest that GsMTx4 may attenuate mechanically induced hyperactivation of these channels and alleviate mechanical hypersensitivity [64]. Recently, GsMTx4, a peptide derived from tarantula venom, has garnered considerable attention as a potent inhibitor acting specifically on the extracellular membrane leaflet to selectively blockade cationic MSCs [61]. Preclinical models validate that GsMTx4 effectively abrogates the mechanical overload induced hyperactivation of these channels, thereby significantly alleviating mechanical hyperalgesia [65]. Consequently, advancing the investigation of specific pharmacological agents like GsMTx4 may forge novel pathways for the precision treatment of tendinopathy, realizing the dual objectives of mitigating nociception at its source and intervening in pathological progression.

#### 2.2.2. ASICs

Historically, research on ASICs focused on neuron‐mediated nociceptive signaling, leading to the assumption that their expression was exclusively neuronal. Subsequent studies challenged this paradigm, confirming ASIC expression across diverse connective tissues, including intervertebral disc (IVD) cells, osteocytes, chondrocytes, and synoviocytes [66–70]. Based on their encoding genes and protein subunits, these channels are classified into multiple subtypes, specifically ASIC1a, ASIC1b, ASIC2a, ASIC2b, ASIC3, and ASIC4. These subunits assemble into homotrimeric or heterotrimeric complexes to form functional ion channels [71]. Their robust expression in sensory neurons suggests a critical role in mediating nociception following tissue acidosis [72–74].

Although tendon‐specific research remains limited, current evidence suggests that ASICs, particularly the ASIC3 isoform, may contribute to nociceptive signaling in tendinopathy [75]. Specifically, extracellular acidification directly gates these channels [71]. Furthermore, Alfredson et al. observed that tendinopathic tissues frequently exhibit relative hypoxia, driving enhanced tenocyte glycolysis, elevated lactate accumulation, and a consequent decline in local pH [76]. The resulting accumulation of protons (H^+^) stimulates ASICs on sensory nerve terminals, serving as a direct mediator of nociception. ASIC3 mediates both transient acid‐evoked currents and sustained steady‐state currents, a dual functionality closely linked to the maintenance of chronic inflammatory pain [77, 78].

Empirical studies demonstrate that nonspecific ASIC antagonists, such as amiloride, significantly attenuate acidosis‐induced chondrocyte apoptosis and preserve cartilage matrix components [79]. Certain NSAIDs directly inhibit ASIC currents, a finding that partially elucidates their therapeutic efficacy independent of classical anti‐inflammatory pathways [80].

#### 2.2.3. Voltage‐Gated Calcium Channels

Magra et al. first demonstrated the functional expression of VOCCs in human tenocytes [51]. This finding indicates that tenocytes can transduce membrane potential fluctuations into intracellular calcium signals. Consequently, VOCCs likely act as mechanotransducers, initiating the conversion of mechanical loads into biochemical cascades. Dysregulation of these channels may thus drive the pathogenesis of chronic tendinopathy, underscoring their potential as therapeutic targets [51]. Table 1 summarizes the mechanotransductive roles of these diverse channels.

**TABLE 1:** Core ion channels involved in tendon mechanotransduction and pain perception, and their pathophysiological effects.

Ion channel category	Representative channel	Primary expression location	Activation conditions	Core downstream effects	References
Mechanosensitive channels	TRPV1	Macrophages	Janus piezoelectric adhesive stimulation	Activates the Ca^2+^/cAMP signaling axis to regulate the macrophage microenvironment and promote tendon‐bone healing.	[81]
	TRPV4	Fibrochondrocytes; mesenchymal stem cells	Mechanical stimulation	Promotes fibrochondrocyte proliferation; mediates calcium signaling to regulate collagen matrix alignment and cellular tension.	[82, 83]
	Piezo1	Tendons; tendon‐derived cells; periosteal progenitor cells	Shear stress; bioreactors; genetic mutations	Promotes tenogenesis: stabilizes cell phenotype and promotes tenogenic differentiation; balances osteogenic/tenogenic plasticity via the YAP pathway; macroscopically regulates tendon tissue stiffness.	[57] [84] [85] [52] [86]
	Mechanogated channels	Injury/healing zone	Blockade by inhibitor (GsMTx4)	After blocking abnormal opening, it inhibits heterotopic ossification and promotes tendon regeneration via the Apelin pathway.	[87]

Acid‐sensing ion channels	ASIC3	Muscle afferents	Ischemia‐ or femoral artery ligation‐induced metabolic microenvironmental shifts	Detects ischemic metabolic perturbations; ASIC3 blockade significantly blunts the exercise pressor and cardiovascular reflexes in murine models of peripheral arterial ischemia.	[88, 89]
	ASIC1a	Afferent neurons	Exercise‐induced metabolic stimuli	Detects the metabolic constituents responsible for eliciting the exercise pressor reflex in healthy murine models.	[90, 91]

Voltage‐gated calcium channels	Calcium channel subunits	Human tenocytes	Mechanical stimuli	Elicits macroscopic whole‐cell currents; implicated as a critical component in the mechanotransductive signaling cascades of connective tissue lineages.	[92]

### 2.3. Immune Response

Historically, tendinopathy was considered a degenerative pathological process. However, recent cellular and molecular research has elucidated the definitive role of molecular inflammation in the pathogenesis of tendinopathy, highlighting it as a pivotal hub for pain generation and maintenance [93, 94]. Unlike typical acute inflammation, tendinopathic inflammation manifests as a low‐grade, chronic, and immune cell‐driven dysregulation of the local microenvironment [11]. Diverse immune cell subpopulations and inflammatory mediators orchestrate this disease progression [95].

#### 2.3.1. Core Proinflammatory Cytokines

Tendon injury triggers an inflammatory cascade. Cytokines and chemokines, released by infiltrating immune cells and activated tenocytes, initiate localized pathological responses. These mediators continuously exacerbate detrimental microstructural and biochemical alterations, ultimately driving the hallmark pathology of tendinopathy [14, 96]. These mediators encompass pro‐ and anti‐inflammatory cytokines alongside diverse growth factors, notably tumor necrosis factor‐α (TNF‐α), interleukin‐1β (IL‐1β), IL‐6, IL‐10, VEGF, and transforming growth factor‐β (TGF‐β). Concurrently, this cascade features the upregulation of cyclooxygenase‐2 (COX‐2) and the synthesis of its catalytic derivative, PGE2 [36, 37, 39, 97–100]. Among the proinflammatory milieu, TNF‐α and IL‐1β emerge as the most potent instigators of chemokine production [101].

The TNF‐α signaling axis is strongly suggested to contribute to tendinopathy pathogenesis. Activated by mechanotransduction, this axis orchestrates ECM structural remodeling and exhibits potent proapoptotic effects on resident tenocytes. In IVD herniation models, TNF‐α accumulation adjacent to nerve roots precipitates ectopic discharges in primary afferent fibers, driving persistent hyperexcitability of nociceptive neurons governing radicular pain [102]. Furthermore, exogenous TNF‐α rapidly provokes mechanical hypersensitivity at the spinal level via distinct presynaptic mechanisms [103]. Intrathecal TNF‐α administration triggers thermal hyperalgesia within 30 min. Mechanistically, TNF‐α binds to receptors on primary afferent terminals, augmenting spontaneous excitatory neurotransmitter release and potentiating dorsal horn neural network excitability, culminating in allodynia [103]. Inflammatory mediators originating from tendinopathic lesions may propagate to the spinal cord via systemic circulation or neural afferents, representing a critical mechanism for pain chronification. Consequently, pharmacological blockade of these cytokine signaling cascades offers a promising therapeutic strategy for managing chronic tendinopathic nociception.

Similarly, the potent proinflammatory cytokine IL‐1β exhibits aberrant upregulation within pathological synovial tissues. This sustained elevation exacerbates localized articular inflammation, subsequently triggering a cascade of proliferative adaptations and progressive degenerative lesions within the tendon [38]. IL‐1β may facilitate substance P release and contribute to neurogenic inflammatory signaling and nociceptive sensitization [104]. Furthermore, IL‐1β drives the downstream induction of critical inflammatory mediators, namely COX‐2 and PGE2 [38], thereby profoundly modulating the tendinopathic pain profile.

#### 2.3.2. Prostaglandins and Nitric Oxide (NO)

Prostaglandins and NO exert a paradoxical, double‐edged regulatory influence in tendinopathy. At physiological concentrations, they orchestrate tissue repair and hemodynamic homeostasis; conversely, under pathological mechanical overload, they pivot into pathogenic effectors driving ECM degradation and nociceptive transmission. Pathological tendon tissues display marked upregulation of pivotal prostaglandin biosynthesis enzymes, including prostacyclin synthase (PGIS), COX‐2, and microsomal prostaglandin E synthase‐1 (mPGES‐1). Concurrently, the pronociceptive neuromodulator N‐methyl‐D‐aspartate receptor 1 (NMDAR‐1) is detected in these lesions [105]. COX‐2, a cardinal biomarker of local tissue inflammation, catalyzes the conversion of arachidonic acid (AA) into prostaglandins like PGE2. This process fuels tendinopathic inflammation by instigating MMP‐dependent ECM catabolism and sensitizing pain signaling [37, 106]. Emerging evidence robustly links NMDAR‐1 activation to nociceptive perception within this context [107]. Intriguingly, PGIS, residing within the identical metabolic cascade, exhibits an inverse functional profile, correlating instead with tendinopathic pain attenuation [107].

Concurrently, NO functions as a critical regulatory molecule in maintaining tendon homeostasis. In vitro assays confirm that judicious concentrations of exogenous NO facilitate tendon repair via augmented collagen synthesis. In contrast, administering NO synthesis inhibitors, such as flurbiprofen, precipitates a reduction in tendon cross‐sectional area and a substantial decay in biomechanical competence [108]. Following tendon insult, the enzymatic activity of NO synthase is markedly upregulated, driving the localized overproduction of NO, an important regulatory factor in the inflammatory milieu. In an exercise‐induced overuse model, NO synthase mRNA expression was excessively upregulated within the supraspinatus tendons of rats subjected to a 14‐day treadmill regimen. Corroborating this, elevated NO synthase expression has similarly been documented in human‐tendon biopsies harvested during shoulder arthroscopy [109, 110].

### 2.4. Neurovascular Coupling Mechanisms

#### 2.4.1. Aberrant Neoinnervation and Neurogenic Inflammation

The healthy tendon proper is profoundly avascular and aneural, with nerve fibers sparsely distributed only in peripheral structures like the loose peritendinous connective tissue [111, 112]. During the progression of painful tendinopathy, the aberrant invasion and ingrowth of sensory nerves into the tendon proper emerges as a highly consistent morphological hallmark. This pathological neural proliferation has been extensively corroborated both clinically and histologically across a spectrum of painful tendinopathic syndromes, including Achilles tendinopathy [17, 30], patellar tendinopathy [31], rotator cuff lesions [113], and lateral epicondylitis [114]. This phenomenon is formally designated as neoinnervation.

Upon these aberrantly infiltrating nerve terminals, the expression of glutamate, substance P, the excitatory NMDAR‐1, and the neurokinin 1 receptor is markedly upregulated [115–117]. Functioning as pivotal neuroinflammatory mediators, glutamate and substance P bind to their respective cognate receptors. This binding activates the immune system, notably mast cells, alongside resident tenocytes and peripheral sensory nerve terminals [118]. Upon activation, mast cells secrete proteases like tryptase, which modulate tenocyte physiology and directly stimulate adjacent nerve fibers. Furthermore, mast cells elaborate nerve growth factor, which subsequently drives the pronounced upregulation of NMDAR‐1, glutamate, and substance P on peripheral nerve terminals. Within this intricate microenvironmental crosstalk, activated tenocytes simultaneously perform a cascading amplification role by actively synthesizing and releasing neuroinflammatory factors, including interleukin‐6, nerve growth factor, and substance P [119]. Subsequently, locally enriched substance P binds to neurokinin 1 receptors to induce peripheral nerve sensitization. This process is coupled with the phosphorylation‐dependent activation of NMDAR‐1, ultimately precipitating and exacerbating localized neurogenic inflammation. [120, 121]. Evidence indicates that substance P upregulates NMDAR‐ 1 and activates it by relieving the voltage‐dependent magnesium block, thereby potentiating its excitability [122]. Concurrently, Kingery and colleagues demonstrated that neurokinin 1 receptor antagonists can reverse these nociceptive dysfunctions [123].

#### 2.4.2. Spatial Coupling of Angiogenesis and Nociception

Ultrasound Doppler imaging and clinical physiological evaluations of patients with Achilles and patellar tendinopathy confirm an aberrant augmentation in capillary blood flow within painful regions. Corresponding histological sections reveal that neovascularization is intimately accompanied by the sprouting of fine neonatal nerve fibers [124]. This spatial coupling of neovascularization and aberrant neural ingrowth directly propagates nociceptive stimuli to the CNS. It also constitutes the primary peripheral structural substrate for the load‐dependent pain characteristic of tendinopathy. Moreover, sensory nerve terminals infiltrating the tendon substance secrete calcitonin gene–related peptide, abbreviated as CGRP. Investigations reveal that this neuropeptide is expressed within tendon fibroblasts, chondrocyte‐like cells, and calcific deposits, implying the existence of a localized neuropeptide reservoir within the tendon proper [125]. In clinical specimens of chronic tendinopathy, the expression of CGRP is demonstrably elevated within sensory nerve terminals [30, 126]. In collagenase‐induced tendon injury models, the dynamic expression profiles of substance P and CGRP strongly correlate with activity‐induced tendon pain. Furthermore, CGRP potentiates the nociceptive and proinflammatory effects of substance P by inhibiting its enzymatic degradation [31].

In summary, a multitude of proinflammatory cytokines, neurotrophins, and neuropeptides, cosecreted by immune cells, tenocytes, and nascent nerve fibers, forge a complex neuroimmune crosstalk within the tendinopathic microenvironment. These mediators precipitate the accelerated degeneration of the ECM while acting as the core molecular machinery driving peripheral sensitization and mechanical hyperalgesia. To conceptually visualize this network, the core neuroimmune mediators, their principal targets, and the associated pathological effects orchestrating the evolution of tendinopathic pain are systematically delineated in Table 2.

**TABLE 2:** Neuroimmune mediators associated with pain in tendon disorders.

Class	Representative molecule	Principal target	Core pathophysiological effect	References
Proinflammatory cytokines	TNF‐α	Primary afferent terminals, spinal dorsal horn	Elicits chemokine release and augments excitatory neurotransmitter release within the spinal cord, rapidly provoking mechanical hypersensitivity.	[101] [103]
	IL‐1β	Tenocytes, neural tissues	Accelerates extracellular matrix (ECM) degeneration, drives COX‐2/PGE2 induction, and facilitates SP release to amplify the nociceptive inflammatory cascade.	[104] [38]

Lipid mediators and gaseous signaling molecules	PGE2	Local tissue matrix and neural elements	Instigates MMP‐dependent ECM catabolism, directly transduces nociceptive signals, and exacerbates localized inflammation.	[37] [106]
	NO	Local microenvironment	Fuels chronic inflammation and nociceptive transmission under pathological overload while orchestrating vascular homeostasis at physiological concentrations.	[108] [109]

Neurotrophins	NGF	Peripheral nerve terminals	Drives aberrant neural ingrowth into the tendon proper and upregulates the expression of nociceptors and algogenic mediators.	[119]

Neurotransmitters and neuropeptides	SP	NK1 receptor	Binds NK1 receptors to induce peripheral neural sensitization; activates NMDAR1, precipitating robust neurogenic inflammation.	[122] [120]
	Glutamate	NMDAR1	Directly mediates nociceptive signal transduction and triggers the inflammatory cascade within local immune cells and resident tenocytes.	[118]
	CGRP	Local microenvironmental targets	Mediates load‐dependent nociception and inhibits the enzymatic degradation of SP, thereby prolonging the nociceptive inflammatory response.	[125] [30]

### 2.5. Central Mechanisms

#### 2.5.1. Central Sensitization

Central sensitization involves altered CNS processing of external sensory stimuli, characterized primarily by the pathological amplification of neural signal transduction. This physiological adaptation significantly enhances the responsiveness of central nociceptive neurons to normal intensity or subthreshold inputs. Historically, tendinopathic pain was considered a strictly peripheral pathological process, leaving central pain mechanisms largely unexplored [127, 128]. However, the clinical manifestation of allodynia and hyperalgesia cannot be adequately explained by peripheral structural damage alone.

Within the nociceptive circuitry, functional neuronal remodeling manifests as substantially potentiated electrical signaling in response to specific sensory afferents. This phenomenon encompasses an elevated frequency of basal spontaneous activity and sustained after discharges following the cessation of noxious stimuli. Collectively, these mechanisms form the biological substrate of central sensitization, driving the pathological amplification of nociceptive signals within the CNS [40, 129]. Furthermore, the expansion of peripheral receptive fields allows hyperalgesia to propagate into adjacent uninjured territories.

The central sensitization inventory (CSI) serves as the primary self‐report instrument in clinical and research settings for identifying and quantifying CS symptoms [128]. Psychometric validation demonstrates the robust internal consistency and criterion validity of the CSI, with a score of 40 routinely utilized as the clinical threshold for identifying significant CS profiles. Facilitated by advancements in QST, researchers can now precisely dissect the multifarious biological pathways underlying individual pain genesis. This modality is broadly applicable across diverse populations and stands as a core metric for evaluating somatosensory sensitivity [18]. Using QST, van Wilgen et al. evaluated central sensitization in cohorts with and without patellar tendinopathy [128]. Significant disparities in pressure pain thresholds exist between asymptomatic athletes and those diagnosed with patellar tendinopathy [130]. Specifically, both mechanical pain and vibration detection thresholds are markedly diminished in patients with patellar tendinopathy. This reduction in mechanical pain thresholds or the presence of pinprick allodynia likely reflects central sensitization mediated by myelinated Aδ fibers.

#### 2.5.2. Plastic Changes in the Motor Cortex

During the progression of chronic tendinopathy, CNS remodeling extends beyond spinal dorsal horn nociceptive sensitization, projecting upward into the network reorganization of the motor cortex [20]. Chronic nociceptive afference and aberrant local load feedback precipitate maladaptive structural and functional neuroplasticity within the cerebral cortex [131]. Crucially, structural plasticity accounts for the chronic persistence of pathological pain states. This encompasses alterations in dendritic spine density, axonal degeneration or regeneration yielding aberrant connectivity, neuronal atrophy [132], and glial proliferation [133].

Transcranial magnetic stimulation (TMS) studies yield compelling electrophysiological evidence substantiating this mechanism. In patients with patellar and Achilles tendinopathy, the primary motor cortex representation governing the affected musculature frequently exhibits topographic smudging. This is accompanied by significantly augmented short interval intracortical inhibition and altered corticospinal excitability [19, 20]. Fundamentally, this cortical inhibition represents a subconscious protective strategy deployed by the CNS to shield the compromised tendon from further mechanical overload. However, chronic cortical inhibition inevitably attenuates higher‐order motor drive to the musculature, culminating in dysregulated muscle recruitment patterns and degraded biomechanical control [134].

The elucidation of this motor cortex plasticity fundamentally revolutionizes traditional tendinopathy rehabilitation paradigms. It delineates why isolated peripheral tissue repair or conventional eccentric loading protocols frequently fail in certain cohorts. Standard resistance training lacks adequate cognitive and cortical engagement to rectify the aberrant inhibition of the primary motor cortex. As highlighted by Rio et al., disrupting the tendinopathic pain cycle necessitates the integration of external pacing cues, such as metronome guided isometric or isotonic contractions. This dual visual and auditory neurointegration recalibrates motor cortex excitability, transitioning the therapeutic focus from peripheral tissue repair toward neuroplastic training aimed at remodeling the corticomotor loop [20].

## 3. Conclusion

In summary, tendinopathic pain is no longer considered merely a localized structural degeneration. Rather, it represents a complex pathophysiological cascade triggered by aberrant mechanical loading and orchestrated by dynamic neurovascular and neuroimmune interactions. This review systematically delineates the pathogenic continuum from mechanotransduction to peripheral sensitization and central modulation. We highlight the pivotal role of MSCs like Piezo1 and TRPV4 as fundamental molecular sensors within this framework. Beyond merely transducing physical forces into biochemical cascades, these channels actively drive metabolic reprogramming and orchestrate the local immune microenvironment via the precise regulation of calcium homeostasis. The hallmark load‐dependent nature of tendinopathic pain fundamentally manifests from the aberrant gating kinetics and impaired desensitization of these channels within the pathological niche.

## 4. Future Perspectives

Future work should link tissue‐level pathological changes with clinically relevant pain patterns. Human‐tendon studies combining quantitative histology or spatial profiling with ultrasound or MRI, QST, and longitudinal functional assessment could clarify whether neurovascular and neuroimmune alterations are associated with persistent pain. Such designs may also help distinguish patients whose symptoms are primarily related to local nociceptive input from those in whom peripheral sensitization or central sensitization plays a larger role.

These uncertainties also limit the immediate clinical translation of pathway‐targeted therapies. Although mechanosensitive channels and neurotrophin pathways are potential targets, direct inhibition of Piezo1, TRPV4, or ASICs, including GsMTx4‐based approaches, remains preclinical or early translational. Similarly, experience with anti‐NGF therapy in osteoarthritis cannot be directly extrapolated to tendinopathy because of joint‐safety concerns. Before these approaches can be considered for clinical use, tendon‐specific studies should establish appropriate delivery strategies, target engagement, preservation of physiological mechanotransduction, and long‐term structural and functional safety.

## Funding

This study was supported by the National Natural Science Foundation of China (Grant No. 81772390) and the Wu JiePing Medical Foundation (Grant No. 320.6750.2020‐03‐07).

## Disclosure

All authors approved the final version to be published.

## Conflicts of Interest

The authors declare no conflicts of interest.

## Data Availability

The data that support the findings of this study are available from the corresponding author upon reasonable request.
